# Fever of Unknown Origin and Hepatitis as the Initial Presentation of Anti-MDA-5 Positive Dermatomyositis: A Case Report

**DOI:** 10.31138/mjr.33.3.361

**Published:** 2022-09-30

**Authors:** Vishal Mangal, Arun Hegde, Jayraj Hasvi, Harikrishnan P, Ankit Kumar, Nidhi Goel, Anil Shankar Menon

**Affiliations:** 1 Department of Internal Medicine, Military Hospital, Ambala, Haryana, India,; 2 Department of Rheumatology, Command Hospital Southern Command, Pune, Maharashtra, India,; 3 Department of Internal Medicine, Armed Forces Medical College, Pune, Maharashtra, India

**Keywords:** dermatomyositis, anti MDA-5, fever, hepatitis, case report

## Abstract

Petersdorf and Beeson first defined fever of unknown origin (FUO) in 1961, and subsequently, over the next 60 years, the definition of FUO has changed considerably. In the western world, non-infectious inflammatory diseases are the most common cause of FUO; however, in developing countries, infections remain the leading cause of FUO. Dermatomyositis (DM) is an autoimmune inflammatory disease of unknown aetiology which mainly affects skin and muscles. Anti-melanoma differentiation-associated protein 5 (MDA-5) positive DM generally presents with classical cutaneous manifestations, early interstitial lung disease, and patients generally do not have clinical features of muscle involvement. We present a case of a 39-year-old male who presented with FUO and hepatitis and was diagnosed as clinically amyopathic DM after two weeks of admission. Subsequently, he was found to have a high titre of Anti-MDA-5 antibody. This is the first case of Anti-MDA-5 positive DM presenting as FUO and hepatitis with a favourable outcome to the best of our knowledge.

## INTRODUCTION

Fever of unknown origin (FUO) means any febrile illness without any obvious aetiology initially. Petersdorf and Beeson first defined fever of unknown origin (FUO) in 1961, and subsequently, over the next 60 years, the definition of FUO has changed considerably. The inclusion of early fluorodeoxyglucose (FDG) positron emission tomography (PET) in the diagnostic algorithm of FUO has remarkably reduced the time to diagnosis.^1^ The most common causes of FUO in adults comprise infections, non-infectious inflammatory diseases (NIID), malignancies, miscellaneous and undiagnosed cases. In the western world, NIID are the most common cause of FUO^2^; however, in developing countries, infections remain the leading cause of FUO.^3^ Among the NIID the most common diseases in Indian studies were systemic lupus erythematosus, vasculitis, mixed connective tissue disorder, and adult-onset Still’s disease.^3,4^

Dermatomyositis (DM) is an autoimmune inflammatory disease of unknown aetiology which mainly affects skin and muscles. It is a rare disease with an incidence of less than one per 100,000 population and presents with classic skin manifestations and symmetrical progressive proximal muscle weakness.^5^ FUO as the initial manifestation of DM is even rare, accounting for only 0.73% of the cases in the study by Zhou G et al. comprising of 1641 patients with FUO.^6^ Anti melanoma differentiation-associated protein 5 (MDA-5) positive DM generally presents with classical cutaneous manifestations like Gottron’s papules, heliotrope rash, palmar papules, mucosal pain, early interstitial lung disease, and patients do not have clinical features of muscle involvement.^7^ To the best of our knowledge, there is only one case reported in the literature of Anti-MDA-5 positive DM presenting as FUO^7^; however, FUO and hepatitis as the initial presentation of Anti-MDA-5 positive DM without characteristic skin lesions and interstitial lung disease (ILD) has never been reported in the literature.

## CASE REPORT

**Figure 1.**
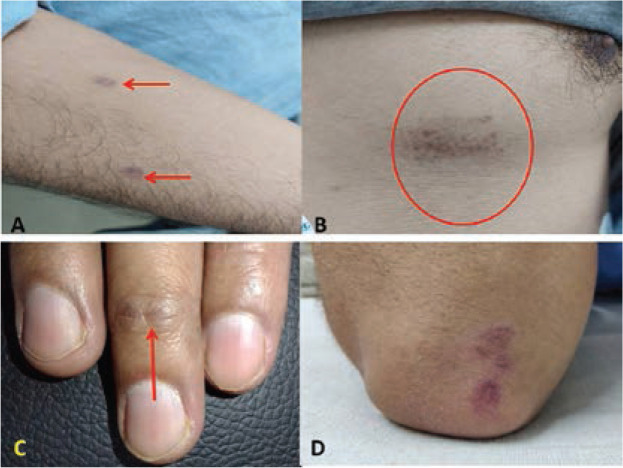
**(a)** Maculopapular erythematous rash with scaling over the forearm. **(b)** Hyperpigmented macular rash over the chest just below the left nipple. **(c)** The red arrow shows Gottron’s papules over the dorsal surface of the distal interphalangeal joint of the right hand. **(d)** A violaceous rash over the right elbow: Gottron’s sign.

**Figure 2.**
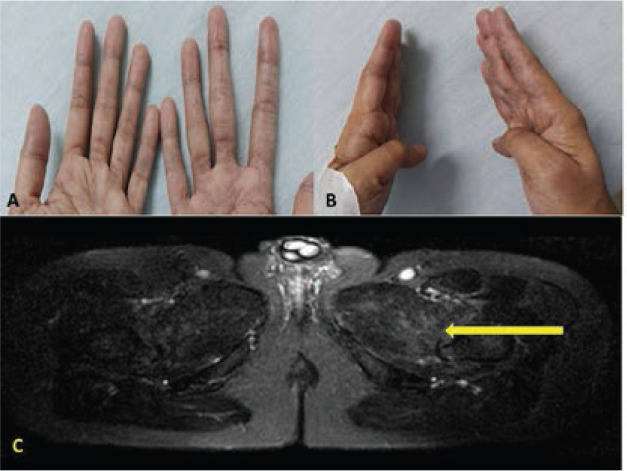
**(a)(b)** The rough and cracked skin over the radial aspect of the index finger with hyperkeratotic lesion and desquamation: Mechanic’s Hands. **(c)** MRI axial T2/short tau inversion recovery: The yellow arrow shows the hyperintense signal in the gluteus minimus, iliopsoas, pectineus, and obturator externus consistent with oedema.

**Figure 3.**
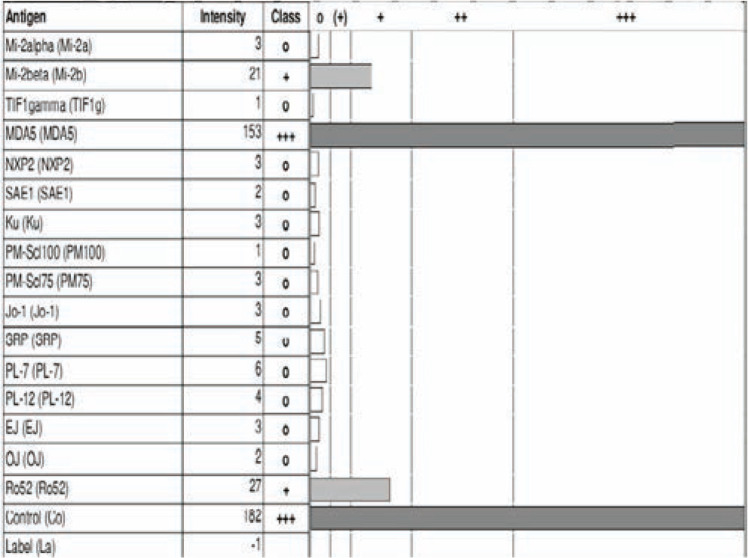
Myositis antibody profile.

**Figure 4.**
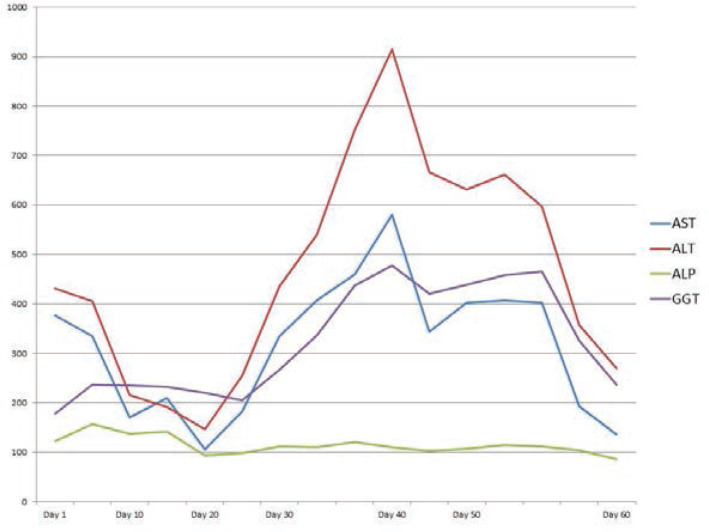
The trend of various liver enzymes from admission to discharge.

A 39-year-old male with no relevant past medical history and family history of pulmonary Koch’s (mother was diagnosed six months back and is on anti-tubercular therapy), presented to our centre with complaints of fever of two weeks duration, malaise, and weight loss of 5 Kg over the last one month. The fever was intermittent, not associated with chills or rigors; however, it had an evening rise of temperature. It was associated with night sweats. He denied any history of vomiting, loose stools, burning micturition, travel, or exposure to pets. On general physical examination, he was febrile with an axillary temperature of 101 degrees F. His blood pressure was 138/92 mm of Hg. He did not have tachycardia or tachypnoea. He had a single right axillary lymph node measuring 1.5 cm in diameter, firm in consistency, mobile, non-tender, not adherent to underlying muscle or overlying skin. He had two macular lesions over the inner aspect of his right forearm (**Figure 1A**) and a macular brown-coloured lesion over the right side of the chest just below the nipple (**Figure 1B**). The rest of the systemic examination was unremarkable. Clinical diagnosis of Koch’s was considered because of prolonged fever, weight loss, night sweats, and family history of Pulmonary Koch’s. The initial laboratory evaluation is presented in **Table 1**. He had microcytic hypochromic anaemia with Mentzer index < 13 and raised aspartate aminotransferase (AST), alanine aminotransferase (ALT), alkaline phosphatase (ALP), lactate dehydrogenase (LDH) levels. Initial fever workup revealed positive IgM typhidot and raised titre (1: 160) of the Weil Felix Test. Because of ongoing fever, transaminitis, and skin rash, the possibility of Enteric fever and rickettsial infection was considered and the patient was administered Ceftriaxone 1 gm intravenous twice a day, along with Doxycycline 100 mg orally twice a day. The patient underwent contrast-enhanced computed tomography (CECT) of the chest and abdomen, which revealed normal lung parenchyma with normal liver and spleen. There was no evidence of any enlarged hilar, mediastinal, or mesenteric lymph nodes on CECT. The patient continued to have fever with trans-aminitis and raised inflammatory markers with negative infective workup (**Table 2**) even after two weeks of hospital admission**.** He underwent whole-body PET, which revealed few discrete mediastinal lymph nodes with mild FDG uptake (Maximum SUV 4.41), likely inflammatory or infective aetiology. At this stage, we considered the diagnosis of Sarcoidosis and other autoimmune connective tissue disorders. However, the patient had normal serum angiotensin converting enzyme levels. We reviewed the history and clinical examination. The patient had developed pain in both the lower limbs, and on examination he did not have muscle tenderness or weakness. He was noted to have a papular rash over the knuckles **Figure 1C** and a purple-coloured macular lesion over the dorsal aspect of the right elbow (**Figure 1D**) with roughening and cracking of the skin over the fingers and palm. He also had painless fissuring of the fingers (**Figure 2A,B**). Because of the Gottron sign, mechanic hands, elevated muscle enzymes, and absence of clinical evidence of muscle weakness the diagnosis of dermatomyositis was considered likely clinically amyopathic dermatomyositis (CADM). The patient was subjected to magnetic resonance imaging of both thighs, which was suggestive of acute myositis (**Figure 2C**). He was administered methylprednisolone 750 mg intravenous for three days followed by oral prednisolone 50 mg daily. Following the pulse glucocorticoid therapy patient became afebrile. A detailed immunological workup was done which revealed normal serum immunoglobulin profile with normal C3, C4 levels. The anti-nuclear antibody was negative by indirect immunofluorescence. He had normal levels of serum anti myeloperoxidase antibody and serum anti proteinase 3 antibody**.** He tested strongly positive for the Anti-MDA-5 antibody (**Figure 3**). Hence the diagnosis of Anti-MDA-5 dermatomyositis was established. The patient underwent muscle biopsy from the right thigh which had essentially unremarkable histomorphology. Skin biopsy from the volar aspect of the right forearm was also done which was suggestive of leucocytoclastic vasculitis. The patient’s AST and ALT showed a declining trend; however, he had a persistent elevation of gamma-glutamyl transferase (GGT). The trend of enzymes over the period of hospitalization is depicted in (**Figure 4**). The patient was evaluated in detail for the cause of elevated ALT and GGT. However, he tested negative for hepatitis A, hepatitis E, chronic or occult hepatitis B, and autoimmune hepatitis. He also underwent Magnetic resonance cholangiopancreatography which was normal. Finally, the patient was subjected to a Liver biopsy which showed some foci of inflammation. Hence the liver damage was attributed to the primary disease, ie, DM.

**Table 1.** Laboratory Workup.

**Parameter**	**On Admission**	**After 2 Weeks**	**After 4 weeks**	**At Discharge**	**Reference Range**
Haemoglobin(g/dL)	11.0	9.3	10.9	9.8	12 to 16
MCV	59.8	62.3	63.8	66.9	
Total leucocyte count (cells/microL)	109100	7300	10400	9200	4000 to 11000
T Bilirubin(mg/dL)	0.6	0.3	0.6	0.6	0.3 to 1
AST(U/L)	377	105	580	135	10 to 40
ALT(U/L)	431	147	914	270	10 to 40
CPK (U/L)	-	265	-	85	55 to 170
ALP	122-	93	121	86	30 to 120
GGT	176	205	477	237	9 to 50
LDH	428	215	273	-	80 to 225
Total Protein(g/dL)	7.7	6.4	6.3	5.8	5.5 to 9
Albumin(g/dL)	3.5	3.0	3.3	2.8	3.5 to 5.5
Globulin(g/dL)	4.2	3.4	3.0	3.0	2.5 to 4.0
Ferritin (ng/ml)	1650	4086	2244	910	8 to 388

MCV: Mean corpuscular volume; AST: Aspartate aminotransferase; ALT: Alanine aminotransferase; ALP: Alkaline phosphatase; LDH: Lactate dehydrogenase; GGT: Gamma-glutamyl transferase.

**Table 2.** Infective workup.

Blood Culture	No growth after five days
Urine Culture	No growth after 72 hours
Dengue NS1Antigen	Negative
Serum Dengue IgM antibody	Negative
Typhi Dot/Serum Salmonella Typhi IgM antibody	Positive
Serum Leptospira Antibody IgG & IgM	Negative
Serum tsutsugamushi (Scrub Typhus) antibody	Negative
Toxoplasma IgG and IgM	Negative
Rubella IgG	Positive
Cytomegalovirus IgG	Positive
Herpes Simplex Virus 1 +2 IgG and IgM	Positive
Brucella Ig M	Negative
Epstein Barr virus antibody to viral capsid antigen IgM	Positive
Weil Felix Test Proteus Antigen OX 19 and OX K	1:160 (Reactive)

## DISCUSSION

Anti MDA-5 DM was first described in 2005 by Shinji Sato et al. when they found a new antibody in CADM patients and named it anti-CADM 140 which later on was identified as Anti-MDA-5.^8^ Subsequently, it was identified that Anti-MDA-5 DM generally presents as CADM in 30–70 % of the patients.^9,10^ The autoantibodies are seen in 50% to 70 % of the patients with DM and out of all the DM patients’ Anti-MDA-5 antibody is seen in only 11 – 13 % of the patients.^9,10^ The median age at presentation is 51–53 years, with female preponderance. Our patient was diagnosed at the age of 39 years and was male. Both the age and sex were unusual for considering the diagnosis of CADM; however, the onset of atypical skin lesion and mechanic’s hands lead us to evaluate for DM.

Anti–MDA-5 DM patients have a distinct mucocutaneous phenotype which is specific to this particular type of DM; however, these patients can also have the typical skin lesions of DM. The cutaneous lesions specific to Anti-MDA-5 DM include erythematous macules and papules over the palmer surface of hands. Palmer papules are usually painful and can lead to substantial morbidity. Skin ulceration is one of the most specific signs of Anti-MDA-5 positive DM seen in 82 % of the patients.^11^ The most common sites of involvement are the pulp of fingers, dorsal surface of interphalangeal joints, elbows, knees, and nail folds. Other characteristic skin lesions include non-scarring alopecia, gum pain, panniculitis, and oral ulcers. Mechanic’s hands which were once considered as a pathognomic feature of antisynthetase antibody-positive DM can also be seen in Anti MDA-5 DM.^12^ Our patient had Gottron’s papules over the distal interphalangeal joint of the middle finger of the right hand (**Figure 2**), along with Gottron’s sign over the right elbow (**Figure 3**). He also had mechanic’s hands and oral ulcers initially. The development of these skin lesions two weeks after admission leads us to consider the diagnosis of CADM in our patient.

ILD is the most common and dreadful systemic complication of Anti-MDA-5 DM because it could progress rapidly and can lead to fatal outcomes. The prevalence of ILD in Anti MDA-5 DM is 93 – 100% in Asians^10, 13^ as compared to 53–73% among patients’ from western countries.^14, 15^ Our patient did not have any clinical symptoms of ILD. However, we did a high-resolution computed tomography (HRCT) of the chest to look for subclinical ILD but there was no evidence of ILD on the HRCT chest also. This was an unusual finding and might be explained by the fact that he was diagnosed at the age of 39 years, and he might develop ILD subsequently. Given the high risk for development of ILD, we plan to screen him for ILD by annual HRCT chest.

Anti MDA-5 DM can also be associated with fatigue and nonspecific constitutional symptoms. The prevalence of fever is estimated to be 46 – 69 %; however, FUO as the initial presentation is rare and has been reported in one case report earlier.^7,16^ Arthritis occurs in 30–82 % of the patients. It is usually symmetrical polyarthritis involving the small joints of hands resembling rheumatoid arthritis. However Rheumatoid factor and anti-citrullinated protein antibodies are usually negative.^11,16^ Our patient presented with FUO; however, he did not have any evidence of arthritis clinically or on imaging.

The anti-MDA-5 DM is associated with a reduced risk of malignancy however the risk still exists. We also screened our patient with tumour markers and whole-body FDG-PET to look for an occult malignancy however he did not have any evidence of malignancy at the time of diagnosis.

The diagnosis of anti-MDA-5 DM can be established by demonstration of Anti-MDA-5 antibodies in the patients’ serum. 90% of the patients have the negative antinuclear antibody, as was the case with our patient. This is because MDA5 is a cytoplasmic protein. Serum ferritin levels are generally elevated in Anti MDA-5 DM and some studies have demonstrated that the level of ferritin correlates with the disease activity and ILD. The ferritin levels come down after successful treatment.^11^ Our patient also had a serum ferritin level of 4086 ng/ml, which decreased to 910 ng/ml two weeks after initiation of glucocorticoids.

Hepatitis marked by elevated levels of AST, ALT, ALP, and GGT has never been reported in the literature as the initial manifestation of DM. However small studies exist which have shown liver damage in patients with DM. A study by Takahashi et al,^17^ showed that 33 % of DM and polymyositis patients had evidence of liver damage directly due to the disease process. Similarly, a recent study by Tatsuhiko Wada et al.^18^ showed that 72.2 % of patients with PM/DM had evidence of liver damage described as ALT level more than two times the upper limit of normal. AST level can be raised in patients with DM however elevation of ALT, and GGT is not expected in cases of DM. Our patient had ALT levels more than 20 times the upper limit of normal and GGT levels were also more than 10 times the upper limit of normal. We evaluated him for all the possible causes like chronic hepatitis B, hepatitis C, autoimmune hepatitis, acute hepatitis A and E. Subsequently we attributed the liver involvement to the primary disease after normal MRCP and liver biopsy. The management of each patient of DM needs to be individualized. Most of the data on management comes from open-label studies, case reports, and retrospective studies. The treatment and the choice of drug depend upon the disease severity, cutaneous manifestations, and presence or absence of ILD. For cutaneous disease, the options available are aggressive photoprotection, antipruritic drugs, antimalarials, and topical anti-inflammatory medications. The systemic therapy includes glucocorticoids, mycophenolate mofetil (MMF), methotrexate, intravenous immunoglobulins (IVIG), and rituximab.^5^ Systemic glucocorticoids are effective for lung and muscle disease but, their efficacy in cutaneous DM is variable. On the other hand, MMF is effective against muscle, lung, and cutaneous disease. IVIG is not used upfront, it is a second-line agent reserved for refractory disease in both skin and muscle. Rituximab is again reserved for refractory muscle and lung involvement. However, for refractory cutaneous disease, its role is not well established. We treated our patient with antimalarials, pulse glucocorticoids, followed by oral glucocorticoids at the dose of 1mg/kg. We pulsed because of hyperferritinemia as the literature suggests that raised ferritin is the marker of severe disease and should be aggressively treated.^19^ We plan to start him on MMF as the glucocorticoid sparing agent.

## CONCLUSION

Dermatomyositis is a rare disease, and Anti-MDA-5 positive DM is even rare. This case highlights the importance of regular clinical examination in the hospitalized patient. The onset of skin lesions even in the absence of muscle weakness made us suspect DM; however, the elevated liver enzymes confounded the initial diagnosis. This case highlights the successful management of the rare presentation of a rare disease.

## ABBREVIATIONS

ALP: Alkaline phosphatase
ALT: Alanine aminotransferase
AST: Aspartate aminotransferase
CADM: Clinically amyopathic dermatomyositis
CECT: Contrast enhanced computed tomography
DM: Dermatomyositis
FDG: Fluorodeoxyglucose
FUO: Fever of unknown origin
GGT: Gamma-glutamyl transferase
HRCT: High resolution computed tomography
ILD: Interstitial lung disease
IvIg: Intravenous immunoglobulin
LDH: Lactate dehydrogenase
MDA5: Melanoma differentiation-associated protein 5
MMF: Mycophenolate Mofetil
NIID: Non-infectious inflammatory diseases
PET: Positron emission tomography
